# Obituary

**Published:** 1883-07

**Authors:** 


### Obituary.

EDWARD F. DOWD, of this city, died June 13th, 1883, at the age of 20. He was born in New York and educated in her public schools. At the beginning of the lecture term in October, 1880, he entered Columbia Veterinary College as a student, completing the course in the spring of 1882, but not receiving his diploma being under age. Soon after he left college he developed symptoms of lung trouble (Pulmonary Tuberculosis), steadily failing in health, although not confined to his bed until a short time before his death. He was hopeful of final recovery until the last few weeks of his life. When convinced that he could not recover he manifested a submissive Christian spirit, quietly and peacefully passing into the sleep of death. His student friends remember him as a genuine attractive companion, full of life and physical vigor. He had a remarkable memory which enabled him to take high rank as a scholar among his associates. Having once heard a thing, and comprehended it, he never forgot it. Had he survived there was every indication that he would have become one of the brilliant lights in the firmament of comparative science.

CHARLES D. HOUSE, D.V.S., died at the residence of his uncle, P. C. House, M. D., in East Bethel, Vermont, on Thursday, April 3, 1883.

Dr. House was a native of Vermont. His early education was limited to such knowledge as could be obtained by irregular and intermitting attendance at a district school.

Early in the war for the Union he enlisted in the volunteer army and distinguished himself as a fearless, brave, and faithful soldier. He participated in the battles fought in the Red River country and at Port Hudson.

He was taken prisoner at one time by the rebels, from whom he afterward escaped by jumping into an almost impenetrable swamp infested with alligators and venemous reptiles. His captors gave him a parting salute of many guns, literally mowing down the flags and bushes around him.

He finally succeeded in reaching the Union lines nearly destitute of clothing, his body terribly lacerated and his feet torn and bleeding. He was afterward honorably discharged from the army, and at the time of his death was a member of Post Baxter, G. A. R., at Newport, Vt.

After his discharge from the army he returned to Vermont and commenced the practice of veterinary dentistry, scoring a pronounced success. Being a man of wonderful nerve and courage, he, in addition to dental skill, soon obtained a noted reputation for his ability in handling and managing vicious horses.

Finding the hills of Vermont narrowing his field of usefulness, he made his way to New York, and very soon commanded the patronage of the best horsemen in the city.

He rapidly obtained a reputation for the ease and certainty with which he subdued and governed horses that were given up as unmanagable.

His success with Mr. Robert Bonner's stallion, Edward Everett, was a notable instance. He had become savage and vicious to that degree that it was almost certain death for an ordinary man to enter his box. "House encountered him alone, and without any show of force, solely by the use of nerve and will power, subdued and controlled the savage animal." On his arrival in New York he was without friends or acquaintances, but he succeeded in a few short years in placing himself at the head of the veterinary profession in this country. His skill in manipulating about the mouth, and his perfect control of the animal, was something wonderful. In many of his specialties he had no equals in his profession. He was the inventor of many valuable and useful surgical instruments. Even the last days of his life were spent in designing and carving models for new and improved apparatus for surgical work. His career as a practitioner was short and brilliant, but it evinced genius of a high order. He marticulated in Columbia Veterinary College in the Fall of 1878, graduating in the class of 1880. He made many warm friends, and had many unpleasant enemies. His sickness was long and painful, though he frequently entertained hopes of ultimate recovery.

In the last days of his illness everything was done for his comfort that kind and sympathetic friends or skillful hands could accomplish.

The cause of death is stated to have been locomotor ataxia, complicated with chronic nephritis. He leaves a wife, one son and two daughters.

"TRIBUTE."

---

DANIEL F. LEAVITT, M. D., died in this city on Saturday, June 19th, 1883.

Dr. Leavitt was one of the Councillors of Columbia Veterinary College. He was interested and active in its organization and support, and did very much to advance the interests of the *Journal of Comparative Medicine* when it was first published. He was a wise counsellor, aud a quiet, faithful friend.

